# Galactomannan antigen detection using bronchial wash and bronchoalveolar lavage in patients with hematologic malignancies

**DOI:** 10.1186/s12941-015-0111-3

**Published:** 2015-11-14

**Authors:** Mahnaz Taremi, Michael E. Kleinberg, Elizabeth W. Wang, Bruce L. Gilliam, Patrick A. Ryscavage

**Affiliations:** Institute of Human Virology and Division of Infectious Diseases, University of Maryland School of Medicine, Baltimore, MD USA; University of Maryland Greenebaum Cancer Center, Baltimore, MD USA; University of Maryland Medical Center, Baltimore, MD USA; The University of Texas MD Anderson Cancer Center, Houston, TX USA

**Keywords:** Galactomannan antigen, Bronchial wash, Bronchoalveolar lavage, Invasive pulmonary aspergillosis

## Abstract

**Background:**

The diagnosis of invasive pulmonary aspergillosis is challenging. It is unclear whether galactomannan (GM) results from bronchial wash (BW) and bronchoalveolar lavage (BAL) samples differ in a clinically meaningful way.

**Results:**

Ninety-six paired (BAL and BW) samples from 85 patients were included. The average age was 53 years, 61 % of the patients were male, and 74.1 % had an underlying diagnosis of AML/MDS (ALL 7.1 %, other hematologic malignancy 18.8 %). 57 (67.1 %) patients were neutropenic, and 56 (65.9 %) patients were receiving mold-active drugs at least 48 h prior to bronchoscopy. The overall agreement between GM detection from BW and BAL was 63.5 % (K = 0.152; 95 % CI 0.008–0.311) and 73 % (K = 0.149; 95 % CI 0.048–0.348) at cut off ≥0.5 and ≥1.0, respectively. Among 43 positive samples, using a GM cut-off of 0.5, 39 (90.5 %) were positive in BW samples whereas 12 (29.3 %) were positive in BAL samples. The median level of GM in BW (0.28) samples was significantly higher than in BAL (0.20) samples among 53 samples with negative results (P = 0.001). There was no statistically significant difference in the median GM values between the BW and BAL samples with positive results (P = 0.08). There was no significant difference in GM detection between samples with positive and negative results with regard to antifungal, beta lactam antibacterial treatment or neutropenia (60.5 vs 56.6 %; 53.9 vs 46 %; 65.1 vs 54.7 %, respectively).

**Conclusion:**

This retrospective study examining two collection techniques suggests that BW may have higher diagnostic yield compared to bronchoalveolar lavage for GM detection.

## Findings

The mortality rate of invasive pulmonary aspergillosis (IPA) remains high in part due to barriers to early diagnosis [1–3]. A definite diagnosis of IPA requires histological evidence of invasive disease or positive cultures from sterile sites [4]. However, respiratory cultures have poor sensitivity and tissue biopsy is not always feasible [5, 6]. Therefore, current diagnostic approaches include non-culture/histopathologic modalities including combination of chest CT imaging and non-invasive, non-culture based tools such as galactomannan (GM) antigen detection in bronchoalveolar lavage (BAL) and bronchial wash (BW) [7–11]. BW samples are defined as the first obtained aliquot of BAL fluid after instillation of small amount of saline into a major airway; BAL samples are collected from the distal bronchioles and alveoli following instillation of a large volume of saline often greater than 140 ml in several aliquots [12]. It is unclear whether GM results from BW and BAL samples differ in a clinically meaningful way. It is hypothesized that bronchial airways may contain more aspergillus hyphae with more subsequent GM release than in alveoli. Alternatively, GM in BAL fluid may be diluted by lavage to an undetectable level by ELISA [13, 14].

In this study, we aimed to compare the performance characteristics of GM antigen tests obtained from BW and BAL samples. This retrospective study included all adult patients with hematologic malignancies at the University of Maryland Greenebaum Cancer Center who underwent bronchoscopy for evaluation of new pulmonary infiltrates while receiving broad spectrum antibiotics or progression of existing lung infiltrates from October 2010 to September 2013. Only patients who had concurrent paired BAL and BW samples separately tested for GM antigen during the same procedure were included. The Platelia Aspergillus enzyme immunoassay (Bio-Rad Laboratories, Hercules, CA, USA) was used to measure GM antigen levels, using different cutoffs for positivity (≥0.5 and ≥1). A patient who underwent repeated sampling in separate bronchoscopic procedures was included in the analysis if bronchoscopy had been performed on the basis of a new pulmonary infiltrate at least 2 months after the first procedure. Plasmalyte solution was not used to perform BAL. Kappa statistics were used to assess the agreement between the GM results obtained from BW and BAL samples. The Wilcoxon test and McNemar’s test were used, as appropriate. All data analyses were performed using SAS V.8.0. 96 paired samples (BAL and BW) were analyzed from 85 patients (AML/MDS 74.12 %, ALL 7.06 % and the others 18.82 %) comprising 52 males and 33 females with an average age of 53 years (range 20–81). 57 (67.06 %) patients were neutropenic (absolute neutrophil count <500//mm^3^) and 56 patients (58.3 %) were receiving mold-active drugs at least 48 h prior to bronchoscopy. Piperacillin-tazobactam was used in nine patients, none had a positive GM test from BAL or BW. Concurrent serum GM assays were performed in only 14 patients (16 %) and three of which were positive at a cutoff ≥0.5. Of these three, all three were positive in samples collected by BW but only one was positive in samples collected by BAL (using OD cutoff value of ≥0.5). Of the 11 negative serum GM results, four were positive by BW samples alone, and one was positive by BAL sample alone; the remainder were negative by both BAL and BW. Of the total four culture-positive BAL samples, one yielded aspergillus fumigatus (with a GM of ≥1 from both BAL and BW), and three yielded penicillium species, of which only one had a positive GM >0.5 from BW sample alone.

The overall agreement between GM detection from BW and BAL was 63.5 % (K = 0.152; 95 % CI 0.008–0.311) and 73 % (K = 0.149; 95 % CI 0.048–0.348) at a diagnostic cut off ≥0.5 and ≥1.0, respectively. Among 43 positive samples, using a GM cut-off of 0.5, 39 (90.5 %) were positive in BW samples whereas 12 (29.3 %) were positive in BAL samples. The GM assay performance for BW and BAL fluid samples for different cut-off OD indexes is shown in Table 1.

**Table 1:** Frequency of positive galactomannan antigen in BAL or BW samples at different cut-off galactomannan OD indexes

Sample, n = 96	BAL+, total	BW+, total	BAL+ or BW+
GM index ≥ 0.5	12 (12.5 %)	39 (40. 62 %)	43 (44.79 %)
GM index ≥ 1.0	10 (10.42 %)	26 (27.08 %)	31 (32.29 %)

*OD* optical density, *BAL* bronchoalveolar lavage, *BW* bronchial washing, *GM* galactomannan

The median levels of GM in BW (0.28) samples were significantly higher than in BAL (0.20) samples among 53 samples with negative results (P = 0.001). Though there was no statistically significant difference in median GM values between the BW and BAL samples with positive results (median OD index of 1.3 for the BW samples vs 3.5 for the BAL samples, P = 0.08), there was a higher number of positive GM results (OD index cut-off of 0.5) only detectable by BW (54.1 %) compared to those detectable only by BAL (9.3 %, P < 0.0001). There was no significant difference in GM detection between samples with positive and negative results with regard to antifungal, antibacterial treatment or neutropenia (60.5 vs 56.6 %; 53.9 vs 46 %; 65.1 vs 54.7 %, respectively).

## Conclusions

To our knowledge only two studies have compared GM antigen detection yield in BAL and BW with conflicting results [15, 16]. Similar to the results observed by Seyfarth et al., our study demonstrated that BW and BAL results are not comparable in terms of detection of GM [15].

The poor concordance between the results of GM detection from the two sample methods in our retrospective single center study suggests that the addition of BW to BAL GM detection enhances positive results or it might be a better single diagnostic sample for GM antigen detection. However, we were unable to establish test specificity/sensitivity due to lack of a gold standard, insensitivity of aspergillus species culture and lack of concurrent serum GM assay (proven and probable cases). Further studies are needed to confirm the complementary role of the BW and to evaluate sensitivity, specificity of GM detection in BW and BAL in IPA diagnosis.

### Availability of supporting data

The data supporting the results of this study are included within this article.

## Abbreviations

GM: galactomannan antigen
BW: bronchial wash
BAL: bronchoalveolar lavage
OD: optical density
